# MicroRNA participates in embryo implantation by modulating endometrial tolerance in sows during peri-implantation period

**DOI:** 10.3389/fendo.2025.1555636

**Published:** 2025-08-05

**Authors:** Wenyuan Li, Xinlin Jia, Xiangyu Mao, Yuanyuan Li, De Wu, Shengyu Xu

**Affiliations:** Animal Nutrition Institute, Sichuan Agricultural University, Key laboratory of Animal Disease-resistant Nutrition, Ministry of Education, Ministry of Agriculture and Rural Affairs, Chengdu, Sichuan, China

**Keywords:** miRNAs, endometrium, hormones, cytokines, immunity

## Abstract

MicroRNAs (miRNAs) are a class of non-coding RNA. MiRNAs affect physiological processes by regulating messenger RNA (mRNA) translation of target genes. The peri-implantation period is the period with the most loss of pig embryos, during which the endometrium provides support for embryo selectivity. The effects of miRNAs during the porcine peri-implantation period include roles in pregnancy recognition, embryo adhesion, maternal vascular support, and immune system modulation. 1) During peri-implantation period, miRNAs regulates the synthesis and secretion of estrogen, progesterone and prostaglandin, and thus plays a role in the process of fetal pregnancy recognition and maintenance. 2) miRNAs regulates the expression of integrin, insulin-like growth factor and their receptors in the embryonic adhesion stage, mediates the formation of tight adhesion and invasion of trophoblast cells, and provides structural support for embryonic development. In addition, miRNAs also acted on retinol-binding protein 4, uterine ferritin, cadhrin, matrix metalloproteinase, fibroblast growth factor and other cytokines, creating a suitable environment for embryo growth. 3) A large number of new blood vessels in the endometrium provide sufficient nutrition for the embryo, miRNAs regulates angiogenesis by acting on angiogenic factors and basic fibroblast growth factors and their participating cellular pathways, thus regulating embryo implantation. 4) miRNAs regulates the maternal immune system to prevent the embryo from being rejected by the immune system due to the presence of paternal antigens (swine leukocyte antigen and so on). Here, we reviewed the regulatory role of miRNAs in the peri-implantation period of embryos, in order to provide theoretical support for reducing the loss of embryos during the peri-implantation period by intervening miRNAs expression.

## 1 Introduction

During each estrous cycle, sows release approximately 20–25 oocytes. However, the litter size at birth is reduced to 10–15 piglets. This reduction is largely attributable to the natural loss of pig embryos, which mainly occurs in two stages: one is the peri-implantation period (10–30 days gestation), where the loss of embryos accounts for approximately 30% of the total gestational loss, and the second trimester (50–70 days gestation), which accounts for approximately 10%-15% of the total gestational loss (1). During the peri-implantation period, the endometrial receptivity was changed due to significant changes in cytokines. Only the synchronous development of endometrium and embryo can ensure a suitable environment for the embryo, and synchronous development starts from the secretion of estrogen by the embryo (2). This article reviews the mechanism of miRNA regulation of embryo implantation in porcine endometrium, aiming to provide a reference for the development of more effective nutritional strategies to regulate miRNA expression and improve the survival of porcine embryos during peri-implantation period.

## 2 Introduction to miRNA

**Figure 1:**
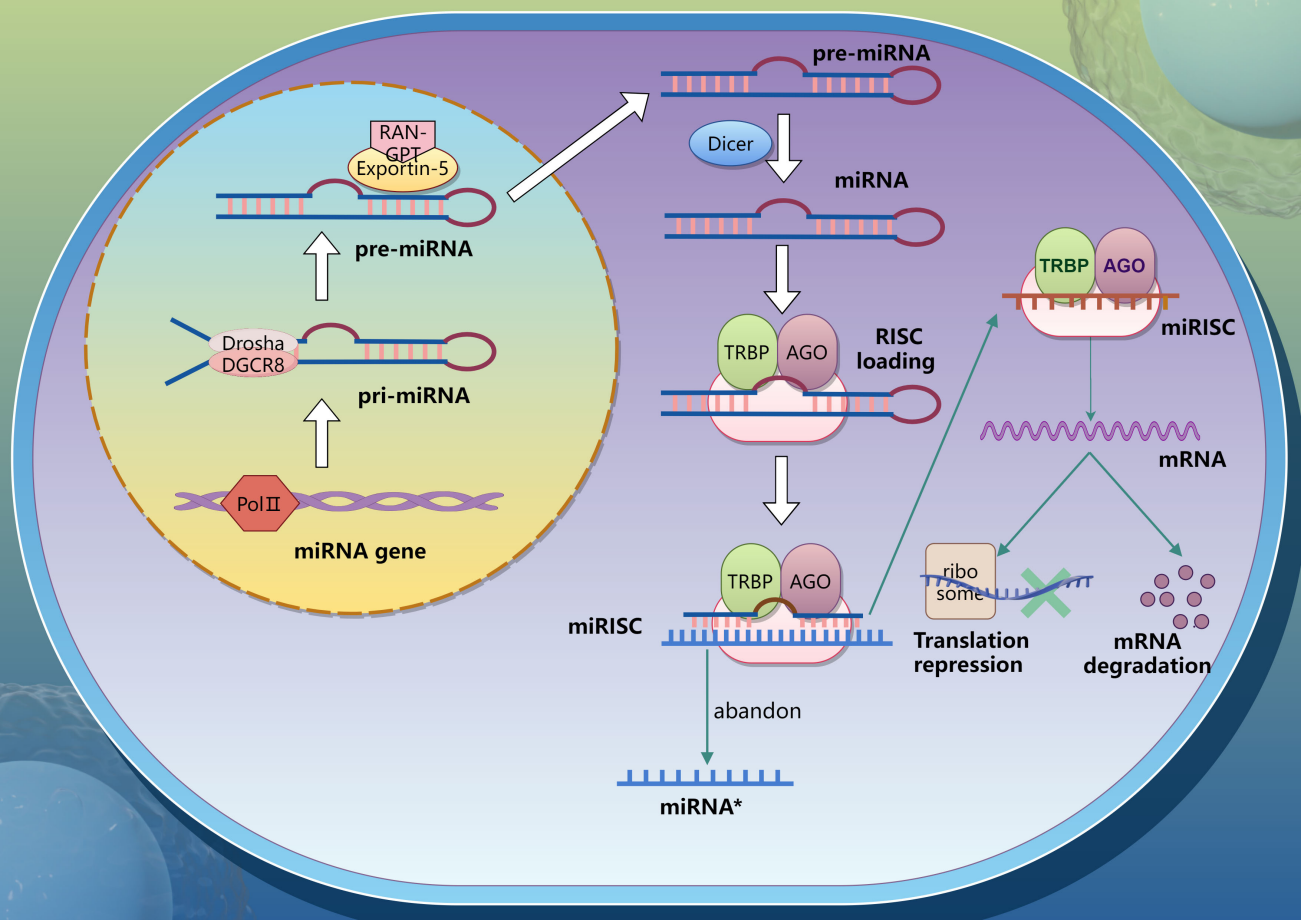
Process of miRNA generation.

MicroRNAs (miRNAs) are a class of non-coding single-stranded RNA with a length of 18–25 nucleotides, which is widely distributed in animals, plants, protozoa and viruses. For most miRNAs, their biosynthesis begins with the catalytic transcription of RNA polymerase II, producing primary miRNAs (pri-miRNAs) with caps and poly (A) tails (3–5). The DROSHA-DGCR8 complex then cleave pri-miRNAs in the nucleus to generate hairpin microRNA precursors (pre-miRNAs). These pre-miRNAs are exported to the cytoplasm through the Exportin-5 and RAN-GTP complexes, and under the action of RNase III enzyme DICER1, the terminal loop is removed to generate a mature miRNA double chain with a size of about 22 bp (6). Subsequently, the double strands were loaded onto the Argonaute (Ago) protein to form miRISC (RISC, RNA-induced silencing complex). When binding to Ago protein, only one chain of Ago protein is stably bound, while the other chain is lost (7). Mature miRNAs degrade mRNA or inhibit its translation by complementary pairing with the 3’untranslated region (3’UTR) of the corresponding mRNA. The mechanism of miRNAs generation is presented in the **Figure 1**.

It has been reported that DICER1 and AGO2 genes involved in miRNA synthesis or transport may have serious effects on reproductive function and embryonic development (8, 9). Stowe H.M. et al. demonstrated that DICER1 is expressed in porcine early embryos and plays a critical functional role (10). Additionally, Krawczynski et al. revealed tissue-specific expression patterns of miRNA biogenesis and transport-related genes in pig embryos, trophoblasts, and endometrium (11, 12). Uterine glands and luminal epithelium are the only sites of DICER1 and AGO2 immune response, and miRNA biosynthesis seems likely to occur in these two sites during early pregnancy in pigs. During days 11–12 of pregnancy, porcine conceptuses exhibited upregulated expression of XPO5, DICER1, TNRC6A, and AGO2, coinciding with blastocyst transformation from spherical to tubular and filamentous morphologies. This period was also marked by elevated transcriptomic activity and increased estradiol (E_2_) secretion. On day 16 of pregnancy, the expression of AGO4 and DGCR8 increased significantly in the endometrium, accompanied by a slight increase in DICER1 and AGO3 (13, 14). In summary, miRNA plays an important role in the regulation of pig peri-implantation period.

## 3 Uterus, endometrium and their receptivity

The uterus serves as the primary site for embryonic growth and development, and consists of three parts: uterine body, cervix, and two separate uterine horns. Endometrium is a dynamic and complex tissue composed of multiple cells targeted by steroid hormones, even during non-pregnancy, and plays a crucial role in the continuation of animals (15, 16). Between day 10 and 13 after fertilization, the endometrium undergoes partial changes in structure and function, and its state changes from a non-receptive state to a receptive state, allowing embryo implantation and pregnancy to begin, which is called endometrium receptivity (17).

The early development of pig embryos is complex. Following fertilization, the zygote forms in the ampulla-isthmus junction of the oviduct and begins cleaving, reaching the two-cell stage at ~26 hours and the four- to eight-cell stage by 48–56 hours, when it enters the uterus. By day 5, the embryo develops into a blastocyst, composed of a blastocoel cavity, trophoblast layer, and inner cell mass. After hatching from the zona pellucida on day 7, the embryo undergoes rapid expansion, growing from 0.5–1 mm (day 7) to 2–6 mm (day 10). Between days 10–11, dramatic morphological remodeling occurs: the spherical embryo (10–15 mm) elongates into a tubular (15 × 50 mm) and then a filamentous structure (1 × 100–200 mm). This transformation maximizes the contact area between the trophoblast and uterine endometrium, facilitating nutrient uptake and supporting subsequent embryo development (18).

Endometrial receptivity is closely related to embryo implantation, and its receptivity will greatly affect the success rate of embryo implantation and further impact embryo implantation. Many embryos have implantation failure clinically, which is related to endometrial receptivity intolerance or insufficient receptivity (19, 20). Endometrial receptivity is affected by a variety of factors, such as hormones, cytokines, angiogenesis, immune factors, etc., and these factors are also regulated by a variety of miRNAs, thus producing interactions, thereby regulating endometrial receptivity and affecting embryo survival.

## 4 Global characteristics of miRNA regulatory networks at the embryo-maternal interface

Recent high-throughput studies have uncovered dynamic miRNA expression profiles at the porcine embryo-maternal interface. During early pregnancy, elongating embryos secrete estrogen (primarily E_2_) as the key signal for maternal recognition. While exogenous E_2_ administration significantly elevates endometrial E_2_ concentrations by day 10 of pregnancy, it does not alter miRNA expression patterns in either the endometrium (21) or blastocysts (22). Comparative studies reveal significant miRNA expression differences in the endometrium between high-fecundity Meishan and Large White pigs during the peri-implantation period. On day 12 of pregnancy, strain-specific miRNAs predominantly regulate p53 and Wnt signaling pathways, which are critical for successful implantation (23). Later in gestation (days 15-50), differentially expressed miRNAs primarily function in endometrial remodeling (including cellular proliferation, migration, apoptosis, cytoskeletal organization, and angiogenesis) and cell communication (particularly hormone response and cell-matrix adhesion) (24).Wessels et al. identified distinct miRNA between maternal and fetal tissues during early pregnancy, with miR-10a, miR-27a, miR-29c, miR-323 and miR-331-5p showing maternal-specific expression patterns potentially linked to early pregnancy loss (25). The maternal immune system undergoes significant adaptation during this period, as evidenced by trophoblast miRNA profiles between days 12–20 that modulate inflammatory processes and cellular immunity. Notably, miR-150, miR-296-5p and miR-19a exhibit were differentially expressed in the endometrium of arrested embryos and healthy embryos on day 20 (26). These findings suggest that miRNAs play an important regulatory role in angiogenesis and immune cell development at the maternal-fetal interface, so they may be essential for embryo survival.

## 5 Interaction between extracellular vesicles and miRNA

Extracellular vesicles are nanoscale particles secreted by organisms, which are encapsulated by phospholipid bilayers and contain components of donor cells (27). EVs can carry different molecules, such as proteins (including common EV markers like CD63 and CD9, along with other cellular proteins), different RNA species (e.g., miRNA), DNA, and membrane-associated components, facilitating intercellular signaling (28). In pigs, EVs were first observed at the maternal-fetal interface on day 16 of pregnancy (29), and have since been isolated from various fluids including uterine fluid and seminal plasma (30). During porcine peri-implantation, active EV secretion occurs in endometrial and trophoblast cells. Notably, miRNA-containing EVs were detected in uterine fluid by days 14-16, demonstrating their role in maternal-fetal communication. For example, uterine cavity-derived EV miR-125b regulates LIF/LIFR expression in endometrial epithelium by day 12 (11). Trophoblast-derived EVs carrying angiogenic miRNAs (miR-16, miR-17-5p, etc.) stimulate endothelial proliferation, suggesting roles in placental vascularization (31). These findings establish EVs as crucial mediators of embryo-maternal crosstalk during implantation.

## 6 MiRNA regulates pregnancy-related hormones and affects endometrial receptivity

**Figure 2:**
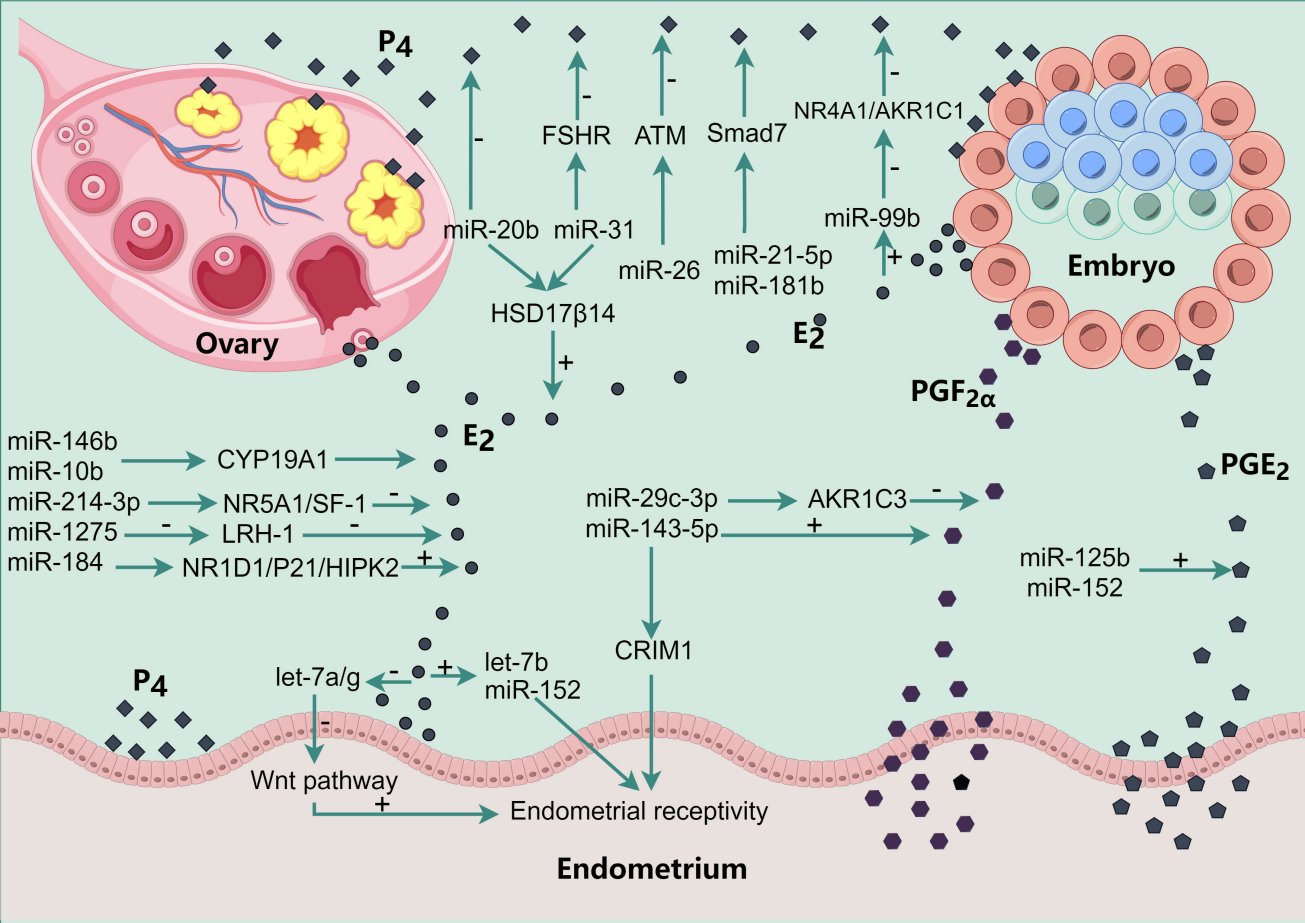
miRNA regulates the production of E2, P4, PGF2α and PGE2 to regulate endometrial receptivity. The various black dots in the figure represent the various hormones secreted by different tissues. The square P4 refers to progesterone, the round E2 refers to estradiol, the hexagonal PGF2α refers to prostaglandin 2α, and the pentagonal PGE2 refers to prostaglandin E2.

The establishment of endometrial receptivity in pigs is tightly regulated by ovarian steroid hormones, including 17β-estradiol (E_2_), progesterone (P_4_), and prostaglandins (PGF_2α_ and PGE_2_). During the narrow implantation window, miRNAs critically contribute to uterine receptivity by modulating the synthesis and signaling of these key hormones (**Figure 2**).

### 6.1 MiRNA regulates E_2_ synthesis

The critical role of embryonic E_2_ secretion in porcine pregnancy recognition was first established by Geisert et al. (1982), who demonstrated that day 12 embryos induce endometrial transcriptome changes through E_2_ signaling (14). This finding was later confirmed by Bazer et al. (2014), showing that embryo-derived E_2_ could regulates key endometrial genes (e.g., up-regulation of PTGS2, FGF7 and can be functionally replaced by exogenous E_2_ administration (32). Notably, Meyer et al. revealed that even cytochrome P450 family 19 subfamily A member 1 (CYP19A1) gene knockout embryos maintain luteal survival until gestational days 27-30 (33).

This raises questions about E_2_ as a pregnancy identification signal. In 2020, Kaczynski et al. studied the live model of false pregnancy and found that the endometrial transcriptomes of the E_2_ treatment group were closer to the endometrial transcriptomes of the 12th day of pregnancy, and the control group was closer to the 12th day of the estrus cycle (34). Thus, it’s confirmed that the effect of E_2_ on the endometrial transcriptome in pigs is consistent with that of embryos at the same time, and confirmed that E_2_ is an important signal for pregnancy recognition in pigs.

During pregnancy establishment in pigs, at the embryo-maternal interface estradiol is secreted both by developing conceptuses and maternal organism. miRNAs have the ability to regulate ovarian E_2_ secretion. Both miR-146b and miR-10b in porcine ovarian granulosa cells (GCs) can interact with the 3 ‘-UTR of CYP19A1 to prevent its translation, thereby regulating the secretion of E_2_ mediated by CYP19A1 (35, 36). In 2020, Shi et al. found that miR-214-3p could inhibit the synthesis of E_2_ in ovarian granulosa cells by targeting NR5A1/SF-1 gene (37). In addition, miR-1275 down-regulates the expression of LRH-1 by directly targeting the 3 ‘-UTR of Liver receptor homolog-1 (LRH-1) of porcine ovarian GCs, thereby inhibiting the interaction between LRH-1 protein and CYP19A1 promoter. The synthesis of E_2_ is then inhibited (38). In 2022, Shi et al. further studied and found that miR-184 promoted E_2_ synthesis in porcine GCs, promoted GCs proliferation and inhibited GCs apoptosis by targeting NR1D1, P21 and HIPK2 respectively (39). miR-31 and miR-20b inhibit the apoptosis of porcine ovarian GCs by targeting the 3’UTR of 17-hydroxysteroid dehydrogenases HSD17β14, thereby increasing the level of ovarian E_2_ (40).

The pig embryo begins to produce E_2_ on the 11th day of gestation, ensuring the maintenance of maternal luteal function, progesterone production and the continuation of endometrial secreting activity. Premature exposure of pregnant sows to exogenous E_2_ (days 9–10 of gestation) results in significant changes in the overall gene expression profile of the endometrium, which may lead to a de-synchronization of the uterine environment and lead to embryo loss at days 15–18 of gestation (41). However, Flöter et al. (21). administered different concentrations of E_2_ orally to sows on the 10th day of pregnancy to investigate its effects on embryos. By detecting miRNAs expression and total estrogen concentration changes in endometrial, it was found that high oral dose of E_2_ resulted in a significant increase in total estrogen in endometrial homogenate. Estrogen stimulation did not affect miRNAs expression in endometrium before embryo implantation. Therefore, it is speculated that long-term estrogen stimulation in the endometrium may lead to estrogen tolerance, so miRNAs expression is not changed.

In mouse and human endometrial epithelium, E_2_ decreased the expression of let-7a, while P_4_ upregulated the expression of let-7a, and let-7a/g enhanced uterine receptivity by inhibiting classical Wnt signaling (42). Mice endometrial epithelial cells and stromal cells were treated with E_2_ and P_4_, and let-7b expression was found to increase, and let-7b was transfected into stromal cells, and the results showed that let-7b inhibited cell proliferation and weakened uterine receptivity during the study period (43). E_2_ and P_4_ treatment of human endometrial cancer cell lines can induce the expression of miR-152, and miR-152 prevents the G1/S transition of cell cycle and inhibits cell proliferation by down-regulating the expression of WnT-1 (Wnt Family Member 1) (44).

In general, these results indicate that miRNAs mediate the effects of E_2_ on endometrial receptivity by influencing cellular proliferation pathways, highlighting their potential role in uterine preparation for embryo implantation.

### 6.2 Regulation of P_4_ by miRNA

Processes such as egg-sperm interactions, early embryo movement within the fallopian tubes, uterine implantation preparation, blastocyst attachment, and successful pregnancy are primarily driven by the steroid hormone P_4_ produced by the corpus luteum, which can also stimulate the endometrial epithelium to produce and secrete nutrient fluid that supports embryo development, implantation, and placenta formation (45).

Pig granulosa cells lose their original morphology after ovulation, and are transformed into luteal cells under the action of luteinizing hormone to form corpus luteum. The expression of progesterone receptor (PGR) in granulosa cells continues from coelomated follicles until granulose-lutein transition (46). It was found that miR-378-3p inhibited the expression of PGR in porcine primary granuleous cells, thus inhibiting the activity of the cells (47). MiR-99b and miR-532 are differentially expressed in corpus luteum during early pregnancy and degeneracy, and they are highly conserved among different animal species. The pregnancy recognition signal E_2_ in pigs up-regulates the expression of miR-99b in luteum, and the overexpression of miR-99b down-regulates the gene involved in luteum degeneration, Nuclear Receptor Subfamily 4 Group A Member 1, NR4A1) and Aldo-Keto Reductase Family 1 Member C1 (AKR1C1) were expressed and P_4_ secretion was increased (48). In porcine ovarian granulosa cells, miR-21-5p acts on SMAD Family Member 7 (Smad7) mRNA to promote cell proliferation, inhibit apoptosis inhibits apoptosis and reduces P_4_ concentration *in vivo*, but the specific mechanism is not clear (49). Smad7 influences Transforming Growth Factor Beta Receptor 1, TGFBR1) stability and blocking the binding of TGFBR1 to Smad2/3 to block the Transforming Growth Factor Beta (TGF-β) signaling pathway. MiR-181b can inhibit the expression of Smad7, activate TGF-β signaling pathway, and inhibit apoptosis of porcine ovarian granulosa cells (50). It was found that miR-378-3p inhibited the expression of Interferon Gamma Receptor 1 (IFNGR1), which plays a role in the apoptosis of luteal cells, but did not affect the expression of IFNGR1 mRNA, thus inhibiting the apoptosis of luteal cells (51). miR-31 can induce apoptosis of porcine ovarian granulosa cells by directly targeting the 3’UTR of follicle-stimulating hormone receptor (FSHR), and reduce the level of P_4_. In addition, overexpression of miR-20b in pigs can also reduce the level of P_4_, but the specific mechanism is still unclear (40). MiR-26b promotes apoptosis of porcine ovarian follicular atretic granulocyte cells by targeting ATM Serine/Threonine Kinase (ATM) (52).

Thus, miRNAs can act on NR4A1 and AKR1C1 to promote the secretion of P_4_, and also prolong cell life and increase the secretion of P_4_ by down-regulating the expression of IFNGR1 and Smad7. However, *in vitro* experiments also showed that miR-31 and miR-26b acted on ATM to promote apoptosis and reduce P_4_ secretion, and miR-20b reduces P_4_ secretion.

### 6.3 Regulation of PGF_2α_ and PGE_2_ by miRNA

Kaczynski et al. (2016) found that PGF_2α_ is involved in pregnancy establishment by promoting endometrial tissue remodeling and angiogenesis and by regulation of genes involved in pregnancy-maternal interactions in pigs during early pregnancy (53). Aldo-keto reductase family 1 member C3 (AKR1C3), also known as prostaglandin F (PGF) synthase. It can catalyze the conversion of estrone and PGD_2_ into estradiol and PGF_2α_, respectively. Studies on human uterine epithelial cells showed that miR-29c-3p could reduce the expression of AKR1C3 and the release of PGF_2α_ (54). Cysteine Rich Transmembrane BMP Regulator 1 (CRIM1) is an indispensable factor in the process of cell polarity, proliferation, adhesion and angiogenesis (55–57). CRIM1 inhibits cell proliferation, cell adhesion and prostaglandin secretion in goat endometrium, thus disrupting normal endometrial receptivity. Targeted miR-143-5p CRIM1 regulates endometrial receptivity, and overexpression of miR-143-5p reduces Secreted Phosphoprotein 1 (SPP1) and Integrin Subunit Beta 3 (ITGB3), ITGB5 expression and increased PGF_2α_ secretion (58).

As a conceptual signal, prostaglandin E_2_ (PGE_2_) participates in the establishment and development of early porcine gestation. In pigs, when the mother is in the period of pregnancy recognition, the endometrium will undergo rapid morphological and functional changes, and the endometrium of the pregnant mother begins to synthesize and secrete PGE_2_ to maintain the corpus luteum and prolong the early life of the corpus luteum, so that the endometrium can accept embryo attachment (59). In addition, PGE_2_ can also act through its receptors in the endometrium to stimulate the expression of PGE_2_ receptor gene (PTGER2) and increase the synthesis and secretion of E_2_, which further leads to the increase of PGE_2_ level through positive feedback (60). Fabova et al. showed that after transfecting pig granulosa cells with miR-125b mimics, the expression level of miR-125-b in the cells increased, which led to an increase in the production of PGE_2_, but a decrease in the production of E_2_ (61). In the same year, Fabova et al. again found that the same results were obtained after transfection of miR-152 analogs into porcine granulosa cells (62).

It can be then indicated that miRNAs are involved in the establishment of endometrial receptivity during the early stage of implantation by regulating the secretion of PGF_2α_ and PGE_2_.

## 7 MiRNA regulates cytokines thus involved in peri-implantation endometrial changes

**Figure 3:**
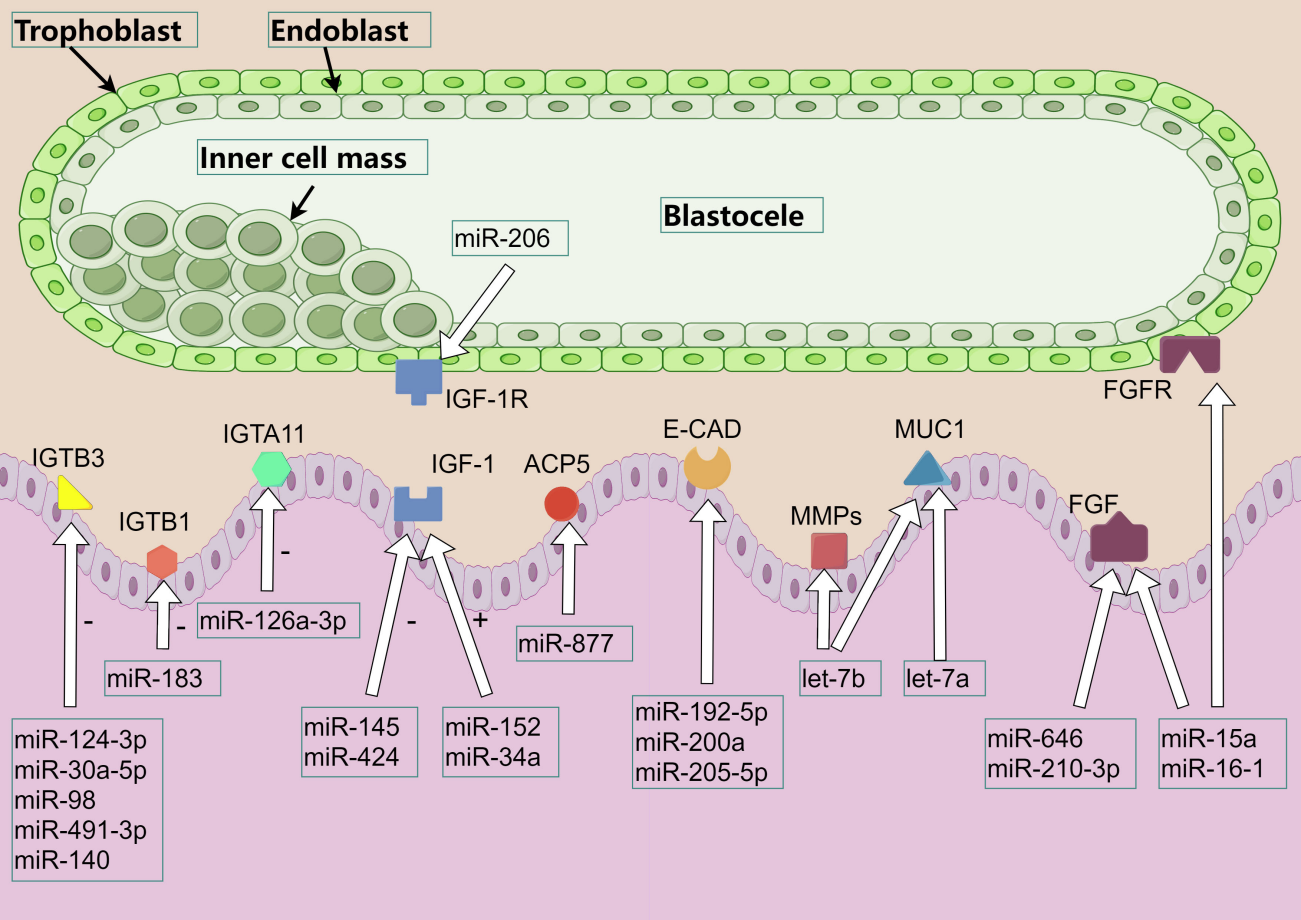
miRNA regulates the involvement of cytokines in endometrial receptivity. The various color patterns in the figure represent different proteins, cytokine, or receptors. Among them, IGTA and IGTB refer to integrin α and β respectively, IGF refers to insulin-like growth factor, IGF-1R refers to insulin-like growth factor 1 receptor, ACP5 refers to acid phosphatase 5, E-CAD refers to E-cadherin, MMPs refers to matrix metallopeptidase, MUC1 refers to mucin 1, FGF / FGFR refers to fibroblast growth factor and its receptor.

The peri-implantation period in pigs begins with the shedding of the embryo’s zona pellucida and the elongation of the trophectoderm, which contacts the maternal endometrial epithelium to determine the site of adhesion. At the established site, microvilli develop between the embryo’s trophectoderm and the endometrium, gradually forming the placenta. This process spans from day 9 to day 26 of gestation. Various cytokines play an important role in this process, and miRNAs participate in the whole process by regulating these cytokines (**Figure 3**).

### 7.1 Regulation of ITGB by miRNA

The process of porcine embryo implantation experienced a dynamic change of adhesion mode. Initially, the initial adhesion was achieved by the interaction between the glycocalyx of trophoblast cells and the endometrial lectin receptor, and then replaced by the integrin beta (ITGB) -mediated trophoblast-maternal extracellular matrix adhesion. This orderly replacement makes embryo implantation more stable. ITGB is a key factor in cell adhesion, and miRNAs can affect embryo adhesion by directly regulating ITGB and its receptors, and embryo adhesion affects the number of piglets to a certain extent. miR-124-3p inhibits the expression of ITGB3 by acting on ITGB3 mRNA, thus inhibiting the proliferation and invasion of endometrium cells. In addition, miR-30a-5p and miR-98 can also act on ITGB3 mRNA to inhibit the invasion of cancer cells. miR-491-3p acts on ITGB3 mRNA to inhibit ITGB3 expression and reduce endometrial receptivity (63–66). miR-140 is involved in inhibiting the migration and invasion of rat endometrial cells by down-regulating the expression of insulin-like Growth Factor 1 Receptor (IGF-IR) and inhibiting the expression of ITGB3 and adhesion plaque kinase (67). The expression of miR-126a-3p was up-regulated at the implantation site of mouse embryos. Bioinformatics prediction and luciferase activity determination confirmed that the target gene of miR-126a-3p was Integrin Subunit Alpha 11 (ITGA11). miR-126a-3p inhibits the translation of ITGA11 mRNA to regulate ITGA11, possibly by impairs the migration and invasion ability of cells, thereby regulating embryo implantation (68). In addition, miR-183 affects cell invasion by regulating ITGB1 in human endometrial stromal cells (69). Thus, miRNAs can regulate cell proliferation and invasion through the expression of ITGB3, IGF-IR and ITGA11 gene proteins, and affect endometrial receptivity.

### 7.2 Regulation of insulin-like growth factor by miRNA

Early in pregnancy, embryonic development requires growth factors and nutrients that are secreted by the uterine epithelium or transported to the mother-fetal interface to promote the attachment of the porcine embryo’s trophoblastic cells to the microvilli of the epithelial epithelium at the apex of the uterus (70). Among these factors, insulin-like Growth Factor (IGF) is thought to play an important role in embryonic and endometrial development. Insulin-like growth factor i receptor (IGF-IR) and Insulin-like growth factor ii receptor (IGF-IIR) are present in pig embryos, and the contents of IGF-I and IGF-II increase in the uterine cavity on the 10th to 13th day of gestation, and IGFs will regulate the attachment of embryos. Studies have found that E_2_ in the early gestation period (days 9-10) can lead to premature breakdown of IGF-binding proteins and a premature decline in the content of insulin-like Growth Factor I (IGF-I) in the uterine cavity, which may affect the attachment and survival of embryos (71). Compared with non-pregnant sows, IGF-IR mRNA expression in endometrium was significantly increased at days 10–12 of gestation, and IGF-I significantly induced AKT Serine/Threonine Kinase 1 (AKT1), extracellular regulated protein kinases (ERK1/2) and Ribosomal Protein S6 (RPS6) phosphorylation, Inhibitor blocking tests have demonstrated that IGF-I collaboratively regulates multiple cell signaling pathways, including intracellular phosphatidylinositol Kinase (PI3K-AKT1-RPS6) and ERK1/2 Mitogen-Activated Protein Kinase (MAPK) signaling pathways. These signaling pathways are essential for the proliferation, migration and survival of trophoblast ectodermal cells in early gestation of pigs (72). The effects of miR-424 on human endometrial cancer cells and IGF-IR inhibit the proliferation and migration of tumor cells (73). On human trophoblast cells, miR-206 inhibits the expression of IGF-I by directly binding to the 3 ‘-UTR of IGF-I mRNA, and miR-206 regulates the IGF-I/PI3K/AKT signaling pathway, affecting the migration and invasion of trophoblast cells (74). miR-145 acts on IGF-IR in mouse endometrium, and overexpression of miR-145 or reduction of IGF-IR will reduce embryo attachment (75). In addition, other studies have shown that the expression levels of miR-152 and miR-34a in porcine granule cells increased after transfection with miR-152 and miR-34a mimics, which led to increased IGF-I production (62, 76).

In short, miRNAs can help embryo adhesion and influence cell proliferation and migration by regulating the expression of IGF-I and its receptor.

### 7.3 Regulation of other cytokines by miRNA

Retinol Binding Protein 4 (RBP4), Acid Phosphatase 5 (ACP5), Cadherin (CAD), Matrix Metallopeptidase (MMPs) and Fibroblast Growth Factor (FGF), as transport protein, transmembrane glycoprotein, extracellular matrix protein and growth factor, plays an important role in implantation and/or pregnancy maintenance in porcine endometrium, respectively. Mucin 1 (MUC1) is a transmembrane glycoprotein that prevents embryo adhesion in the endometrium, negatively impacting embryo implantation.

RBP4 is A transporter of vitamin A, and overexpression of RBP4 in porcine granulosa cells can cause changes in the expression of 17 miRNAs. miRNAs with altered expression are mainly involved in chemokine signaling pathway, peroxisome proliferator-activated receptor signaling pathway, insulin resistance pathway, nuclear factor-κB signaling pathway and steroid hormone biosynthesis (77). ACP5, a metalloprotein secreted by endometrial glandular epithelium, facilitates iron transport to developing embryos. While its direct role in porcine reproduction requires further study, miR-877 has been shown to inhibit cell viability and migration in human cancer models by targeting ACP5 and suppressing the PI3K/AKT pathway (78).CAD is a medium of adhesion between the embryo and endometrial epithelium, and its increase after ovulation and its optimal effect during implantation stage is a sign of uterine receptivity change (79). In murine studies: miR-192-5p maintains epithelial polarity by stabilizing E-cadherin, preventing epithelial-mesenchymal transition (80). miR-200a directly regulates E-cadherin to modulate cell adhesion (81). miR-205-5p enhances endometrial receptivity via ZEB1 inhibition and E-cadherin upregulation (82). MMPs is an enzyme that can degrade collagen, laminin, fibronectin, elastin and proteoglycan in extracellular matrix. MMPs produced by endometrial and trophoblast cells degrade the extracendometrial matrix and promote embryo implantation. Let-7b demonstrates conserved regulatory function in mice, where its overexpression in pregnancy day-7 stromal cells suppresses MMP-9 and its inducer Basigin (Bsg), preserving matrix integrity (43). FGF is a pluripotent growth factor. FGF4 secreted by pig embryo/endometrium activates FGF receptor on trophoblast ectodermal cells, transmits signals through PI3K/AKT pathway, and regulates cell migration (83). miR-210-3p: Limits trophoblast invasiveness by targeting FGF1 (84). miR-15a/16-1: Inhibits angiogenesis via FGF2/FGFR1/VEGFA/VEGFR2 suppression (85). miR-646: Blocks metastatic pathways by downregulating FGF2 (observed in osteosarcoma models) (86). MUC1 inhibits cell-cell and cell-matrix adhesion by blocking receptor-ligand interactions at the cell surface through spatial sites, and the expression of MUC1 is reduced at embryo implantation. Murine studies confirm let-7a/let-7b promote implantation by directly suppressing Muc1 expression, thereby enabling embryo-endometrium contact (87).The regulation of miRNAs on the above cytokines is shown in **Table 1**.

**Table 1:** The regulation of miRNA on cytokines.

miRNA	Gene / Pathway	Influence	Reference
miR-877	*ACP5/* PI3K/AKT	Inhibited cell viability and migration	(78)
miR-192-5p	*E-CAD*	Prevent epithelial-mesenchymal transition	(80)
miR-200A	*E-CAD*	Regulate cell adhesion	(81)
miR-205-5p	*E-CAD*	Promote embryo implantation	(82)
let-7b	*MMP9*/BSG	Prevent the degradation of extracellular matrix	(43)
miR-210-3p	*FGF1*	Inhibit the proliferation and invasion of trophoblast cells	(84)
miR-15a,miR-16-1	*FGF2,FGFR1,VEGFA,VEGFR2*	Inhibit the formation of blood vessels	(85)
miR-646	*FGF2*	Inhibition of cell proliferation, migration and invasion of cells.	(86)
let-7a,let-7b	*MUC1*	Promote embryo implantation	(87)

ACP5, Acidphosphatase5; PI3K, Phosphatidylinositol-3-kinase; AKT, Proteinkinase-BCAD, 853 Cadherin; MMPs, Matrixmetallopeptidase; BSG, Bsgprotein; FGFR1, Fibroblastgrowthfactor 854 receptor1; VEGFA, VascularendothelialgrowthfactorA; VEGFR2, Vascularendothelialgrowth 855 factorreceptor2; MUC1, Mucin1.

## 8 MiRNA regulates endometrial vascular development during peri-implantation period and regulates embryo implantation

**Figure 4:**
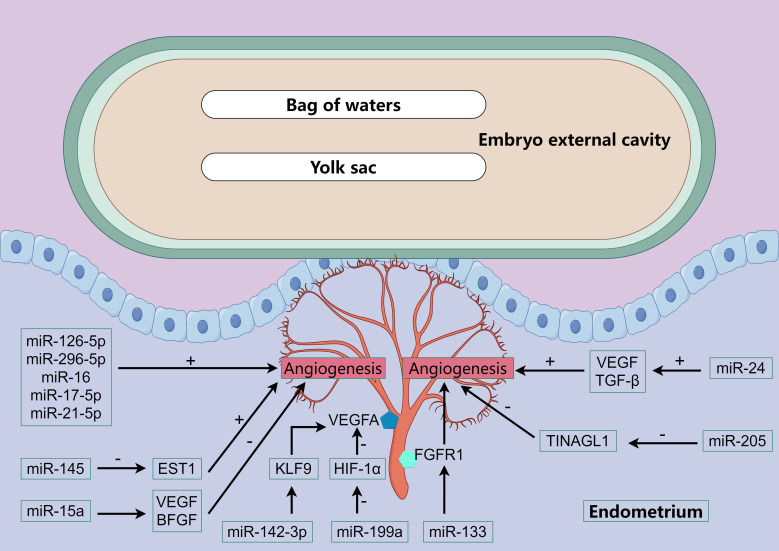
miRNA regulates vascular development. The color polygons in the figure and the various cytokines or receptors that appear. We all have been explained above.

The study of the uterus during the peri-implantation period of pig pregnancy showed that the natural stagnation of pregnancy can be identified by the decrease of blood vessels in the endometrial implantation site and the retardation of embryonic development on the 20 th day of early pregnancy. Comparing gene expression of angiogenic factors and cytokines in the endometrium, endometrial lymphocytes, and trophoblast cells at the attachment sites of normal and stagnant pregnancies revealed reduced expression of Vascular Endothelial Growth Factor (VEGF) and Hypoxia Inducible Factor 1 Subunit Alpha (HIF-1α) in the endometria of stagnant pregnancies (88). In addition to these factors, Basic fibroblast growth factor (BFGF) and Placental Growth Factor (PlGF) also influence blood vessel formation. miRNAs regulates the expression of these factors and is involved in endometrial vascular development (**Figure 4**).

Bidarimath et al. found that exosomes secreted by embryonic trophoblast cells of swine at the 20th day of gestation could promote the proliferation of endometrial cells and the formation of blood vessels (31). miRNAs associated with angiogenesis (miR-126-5p, miR-296-5p, miR-16 and miR-17-5p) were detected in exosomes and were relatively abundant. In addition to these several miRNAs that have been recognized to promote angiogenesis, miR-21-5p has been found in human venous endothelial cells to promote cell proliferation, migration and wound healing, and it has been further confirmed in chicken embryos that miR-21-5p can promote angiogenesis (89, 90). Further studies have found that miR-21-5p promotes angiogenesis by mediating the ERK-MAPK signaling pathway (91). In human endometrial cells, miR-142-3p directly targets KLF9 (Kruppel Like Factor 9) and regulates VEGFA expression (92, 93). Blocking BFGF receptor or PI3K/AKT signaling in adipose stem cells can increase the level of miR-145, which can inhibit ETS1 (ETS Proto-Oncogene 1) expression and promote collateral vessel formation in ischemic mice hind limbs (94). In mice fed a high-fat diet, the ability of bone marrow mesenchymal stem cells to promote angiogenesis is reduced, which is caused by miR-15a inhibiting the expression levels of VEGF and BFGF, and inhibition of miR-15a can restore the ability of mesenchymal stem cells to promote angiogenesis (95). In addition, FGFR1 signaling plays an important role in the tubular formation of endothelial cells induced by BFGF, and miR-133 can inhibit angiogenesis by inhibiting FGFR1 expression (96). miR-24 can promote the survival and angiogenesis of human endothelial cells, which is achieved by promoting the expression of VEGF and TGF-β (97). In studies of hepatoma cells, miR-125b inhibited the expression of PlGF and thus significantly inhibited the angiogenesis of cancer cell lines (98). Other studies have shown that miR-199a can inhibit the angiogenic potential of human endometrial mesenchymal cells, which is partially achieved by inhibiting the HIF-1α/VEGFA pathway (99). In addition, previous studies have shown that renal tubulointerstitial nephritis anti-original 1 (TINAGL1) is a positive regulator of angiogenesis, which can increase endothelial cell invasion and angiogenesis germination. However, miR-205 can inhibit the expression of TINAGL1 gene by directly targeting the 3’UTR of TINAGL1, thereby inhibiting the angiogenesis of porcine endometrial and ultimately affecting the development of embryos after implantation (100).

Brief sum-up, miRNAs mainly affect the ERK-MAPK signaling pathway and the expression of KLF9, ETS1, FGFR1 regulate the generation of VEGF and BFGF to regulate angiogenesis and thus regulate embryo attachment. Whereas miR-125b, miR-199a and miR-205 are miRNAs that inhibit angiogenesis.

## 9 MiRNA regulates immune-related factors to regulate embryo implantation

During the peri-implantation period, the embryo carries paternal antigens (Swine leukocyte antigen, SLA and Y chromosome-encoded male-specific histocompatibility antigen, H-Y antigen) that stimulate the mother’s immune system (101). If the immune system remains intact, the embryo will be killed due to immune rejection, but the reality is that the embryo can still survive because the mother’s immune system is partially shielded. During pregnancy, natural killer cells (NK) and T cells from the mother are recruited into the endometrium, and NK cells in pregnant sows are three times higher than in non-pregnant sows, and T cells are mainly involved in the establishment of the placenta (102, 103).

Chemokines act as signaling molecules during pregnancy, alerting the maternal immune system to local immune responses at newly formed endometrial blood vessels and recruiting immune cells to the implantation site, which can disrupt endometrial angiogenesis and lead to fetal death. Previous studies have shown that porcine chemokines are involved in the recruitment of these immune cells and the establishment of the immune tolerance environment *in utero*. Cysteine-X-cysteine motif chemokine ligand 2 (CXCL2), CXCL5, CXCL11, and CXCL12 are chemokines required for immune cell recruitment, and CXCL9 and CXCL10 are involved in the establishment of the immune tolerance environment of swine uterus (104). CXCL9, CXCL10, CXCL11, and their receptor CXCR3 are localized in stromal cells, endothelial cells, or vascular smooth muscle cells of the swine endometrium and are most richly expressed on day 15 of gestation. Interferon-γ (IFN-γ) can increase the abundance of CXCL9, CXCL10 and CXCL11, and promote the migration of NK and T cells (105). Dual-luciferase reporter gene detection results confirmed that miR-9 can target CXCR4 and CXCL11 in pigs, indicating that miR-9 may play a role in recruitment of immune cells during embryo implantation by regulating the expression of CXCR4 and CXCL11 genes (106). In addition, CXCL12 regulates the aggregation of white blood cells into the abdominal cavity and tissue growth in endometriosis, and 35 miRNAs expression changes were found in endometrial stromal cells treated with CXCL12. The target genes of these miRNAs are mainly involved in chemokines of immune cells, inflammatory and immune responses, and pathological processes of human endometriosis lesions (107).

Around 12 days of swine gestation, the embryo elongates and releases Interleukin-1 beta (IL-1β), and after elongation, interferon IFN-γ and small amounts of IFN-δ are released, which play an important role in the embryo’s attachment to the uterus. In addition to these pro-inflammatory factors, there are IL-6, IL-18, leukemia inhibitory factor (LIF) and Tumor necrosis factor-α (TNF-α), These pro-inflammatory factors create a pro-inflammatory microenvironment in the endometrium, which can receive additional tissue nutrients to nourish the pre-implantation embryo, endometrial estrogen and prostaglandin synthesis. IL-1β can activate p38MAPK and ERK1/2 signaling pathways to promote epithelial cell proliferation (108). IFN-γ is thought to promote the structural changes of endometrium during implantation by regulating the expression of tight junction protein ZO-1 in swine uterine surface epithelial cells (109). Receptors for IL-6 and LIF were expressed in both embryonic and uterine surface epithelial cells of porcine on days 10–14 of gestation, indicating that these cytokines play an important role in the implantation process (110, 111). The pro-inflammatory cytokine IL-18, released from the pig endometrium at 15–18 days gestation, may stimulate the release of IFN-γ in the embryo (112). MiRNAs can regulate the maternal immune system during the peri-implantation period by regulating the expression of these pro-inflammatory factors.

In LPS-induced bovine endometritis, miR-193a-3p exacerbates inflammation by upregulating pro-inflammatory cytokines (IL-1β, IL-6, TNF-α) (113). Conversely, several miRNAs exhibit anti-inflammatory properties: miR-488 and miR-26a suppress IL-1β, IL-6, and TNF-α expression (114, 115). miR-148a specifically inhibits IL-1β and TNF-α production (116). miR-424-5p and miR-24-3p attenuate LPS-induced inflammation by blocking cytokine secretion (IL-1β, IL-6, IL-8, TNF-α) and inactivating the NF-ĸB pathway, with miR-24-3p showing conserved effects in murine models (117, 118). The anti-inflammatory action of miRNAs extends to: miR-643, which reduces IL-1β/IL-6 secretion and NF-κB activation in human endometrial epithelial cells (119). Exosomal miR-218 from inflamed bovine endometrium, which maintains immune homeostasis by suppressing IL-6, IL-1β, TNF-α, and chemokines (MIP-1α/β) (120). Notably, IFN-γ-treated human endometrial mesenchymal stem cells secrete exosomes containing differentially expressed miR-150-5p and miR-196b-5p, which participate in IL-6/8/12 signaling transduction (121).

The above research results indicate that, miRNAs can affect embryo implantation by regulating CXCR4, CXCL11, IL, TNF-α and other immune-related factors. However, the regulation of miRNAs on the expression of these pro-inflammatory factors in porcine endometrium has not been reported.

## 10 Summary and prospect

In conclusion, miRNAs regulate endometrial receptivity during the peri-implantation period in pigs by modulating the secretion of hormones such as E_2_, P_4_, PGF_2α_, and PGE_2_. By regulating the expression of ITGB, IGF, RBP4, ACP, CAD, MMP and FGF, the embryo adhesion process was affected. MiRNAs are differentially expressed at the implantation site of the embryo, which strictly controls the formation of blood vessels and thus regulates the implantation of the embryo.

However, it is very regrettable that there are few studies on miRNAs in pig immune system shielding so far. In the future, we can study the precise regulation and expression of miRNAs in the immune system in many fields such as human conception and animal reproduction, so as to promote the high expression of miRNAs conducive to embryo survival, reduce the occurrence of fetal diseases after pregnancy and delivery, improve the litter size and embryo survival rate of livestock and poultry, and provide a basis for human and animal embryo health.
